# A pre-B acute lymphoblastic leukemia cell line model reveals the mechanism of thalidomide therapy-related B-cell leukemogenesis

**DOI:** 10.1016/j.jbc.2024.107578

**Published:** 2024-07-17

**Authors:** Malvika Ramani, Rishi Kant Singh, Saurabh Shrivastva, Louis Ribeyron, Sanjeev Kumar Gupta, Anita Roy

**Affiliations:** 1 Kusuma School of Biological Sciences, Indian Institute of Technology, New Delhi, India; 2 Faculty of Sciences and Engineering, Sorbonne Université, Paris, France; 3 Dr B R A- IRCH, All India Institute of Medical Sciences, New Delhi, India

**Keywords:** IKZF1, thalidomide, B-ALL, stemness, fibronectin adhesion, B cell differentiation

## Abstract

Lenalidomide, a thalidomide derivative, is prescribed as maintenance therapy for multiple myeloma (MM). Patients with MM receiving lenalidomide were found to develop a distinct therapy-related B cell acute lymphoblastic leukemia (B-ALL). However, the molecular mechanism by which lenalidomide drives B-ALL is unknown. We show that thalidomide treatment of B cell lines increased CD34 expression and fibronectin adhesion. This resembled the effects of *Ikzf1* loss of function mutations in B-ALL. IKZF1 is a transcription factor that can act as both a transcriptional activator and a repressor depending upon the target loci. In our experiments, thalidomide-induced degradation of IKZF1 increased the expression of its transcriptional repression targets *Itga5* and CD34 explaining the increased adhesion and stemness. Strikingly, withdrawal of thalidomide lead to the mis-localization of IKZF1 to the cytoplasm. Moreover, chromatin immunoprecipitation data showed a long-term effect of thalidomide treatment on IKZF1 target loci. This included decreased chromatin occupancy at early B cell factor 1 (EBF1) and Spi1 (PU.1). Consequently, B-cell lineage specifying transcription factors including Pax5, Spi1 and EBF1 were downregulated even after 7 days of thalidomide withdrawal. Our study thus provides a molecular mechanism of thalidomide-induced B-ALL whereby thalidomide alters the chromatin occupancy of IKZF1 at key B-cell lineage transcription factors leading to a persistent block in B-cell differentiation.

Lenalidomide, a thalidomide analog is prescribed as an immunomodulator to transplant eligible patients with multiple myeloma (MM) (1). However, lenalidomide maintenance therapy in MM is associated with an increased risk of therapy-related B-cell acute lymphoblastic leukemia (B-ALL) (2). This second primary malignancy arises from clones genetically distinct from that of MM (3, 4). Although a high incidence of *TP53* mutations has been reported in lenalidomide-associated B-ALL, the molecular mechanisms leading to such transformation are unknown. Thalidomide and its analog lenalidomide are known to induce the mistargeting of Cullin-RING E3 ubiquitin ligase to IKZF1, IKZF3, and SALL4 leading to their degradation (5, 6). Of these, IKZF1 is an important transcription factor of the early B-cell lineage and its absence or haploinsufficiency is associated with poor prognosis in B-ALL (7). IKAROS (IKZF1) is the founding member of a family of transcription factors characterized by the presence of N-terminal zinc finger domains responsible for DNA binding and C-terminal zinc fingers for dimerization (8). Conditional deletion of *Ikzf1* results in a block in the B-cell differentiation at the pre-B cell stage that is characterized by increased cell adhesion (8). Moreover, deletion of the N-terminal zinc finger domains that abolish the DNA binding activity while retaining the dimerization potential (dominant negative effect) also induced a similar differentiation arrest with a concomitant increase in integrin-dependent cell adhesion (9). Therefore, we asked if thalidomide treatment could alter the differentiation program of B-cells, thereby aiding B-ALL transformation.

Using retroviral transduction of *Ikzf1* deletion exon 4 to 7 (IK6) in pre-B acute lymphoblastic leukemia cell line (JM1), we developed a cell line model that recapitulated the phenotype of increased adhesion and stemness as observed in B-ALL mouse models and patient samples (10, 11). We then treated the JM1 parent cell line with thalidomide and observed a similar pattern of increased integrin-dependent cell adhesion and stemness. Strikingly, withdrawal of thalidomide followed by culture of the cells in thalidomide-free media showed a long-term effect on IKZF1-dependent gene expression. This included crucial B-cell transcription factors that were repressed even after 7 days of withdrawal from treatment. Taken together, our study highlights the long-term effects of thalidomide treatment on IKZF1-dependent transcription and B-cell specification.

## Results

### JM1 IK6 recapitulates the B-ALL blast phenotype of increased stemness and adhesion

**Figure 1:**
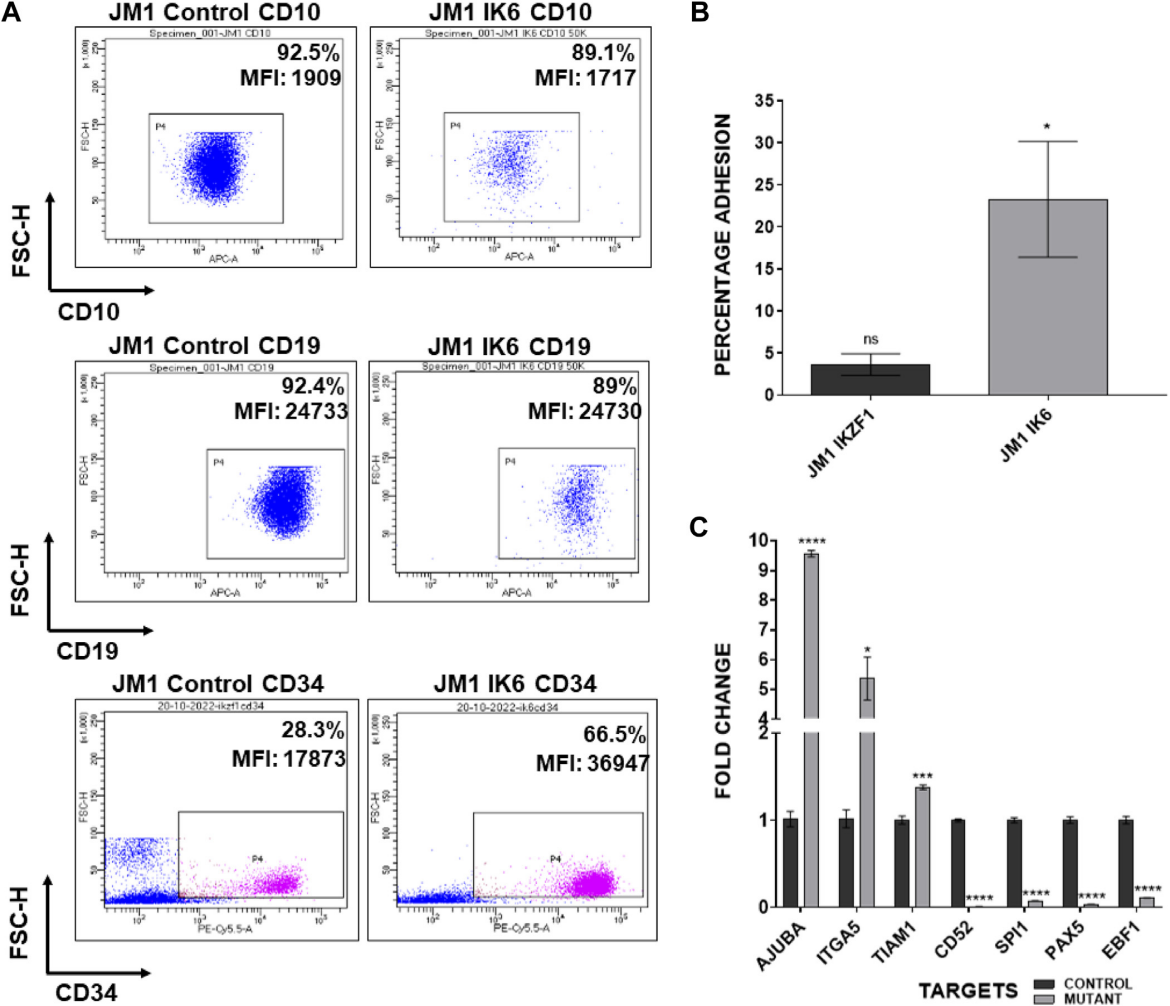
**JM1 IK6 recapitulates the effects seen in B-ALL blasts.***A*, flow cytometry analysis of CD10, CD19 and CD34 levels in JM1 control cells and JM1 electroporated with IK6 expression vector. Percentage of parent population and MFI (Median Fluorescence Intensity) is indicated. *B*, JM1 IK6 (retroviral transduced and sorted) cells showing increased adhesion to fibronectin in comparison with JM1 cells. *C*, qRT-PCR data showing the fold change in expression of adhesion-related molecules *jub*, *itga5*, and *tiam1*, lymphocyte marker CD52, and B cell transcription factors *spi1* (PU.1), *pax5* and *ebf1* determined by quantitative PCR in JM1 IK6 cells compared to JM1 cells. Data has been normalized against the expression of PPIA. (ns: not significant *p* > 0.05, ∗*p* ≤ 0.05, ∗∗*p* ≤ 0.01, ∗∗∗*p* ≤ 0.001, ∗∗∗∗*p* ≤ 0.0001).

We transduced the pre-B ALL cell line JM1 with the dominant negative IK6 construct (12). The aim was to create a cell line model that recapitulated the effects of IK6 or similar loss of function mutations of *Ikzf1* in B-ALL blasts. We observed that compared to the control JM1, JM1 IK6 cells showed increased expression of CD34 without any significant change in the expression of CD10 and CD19 (Fig. 1*A*). JM1 IK6 cells displayed increased adhesion to fibronectin (Fig. 1*B*) when compared to the control cells. Furthermore, the expression of *Itga5* responsible for fibronectin adhesion was observed to increase in the presence of IK6 (Fig. 1*C*). Additionally, JM1 IK6 showed altered gene expression signatures reminiscent of B-ALL blasts including increased expression of cell adhesion genes JUB and TIAM1 and decreased expression of B cell differentiation markers EBF1, Pax5, Spi1 (PU.1) and CD52 (Fig. 1*C*). Interestingly, CD52 is expressed in circulating B cells and is a late-stage B cell differentiation marker (13). Thus, the JM1 IK6 cell line model recapitulated the B-ALL blast phenotype of increased cell adhesion and stemness.

### Thalidomide withdrawal induced mistargeting of IKZF1 to the cytoplasm

**Figure 2:**
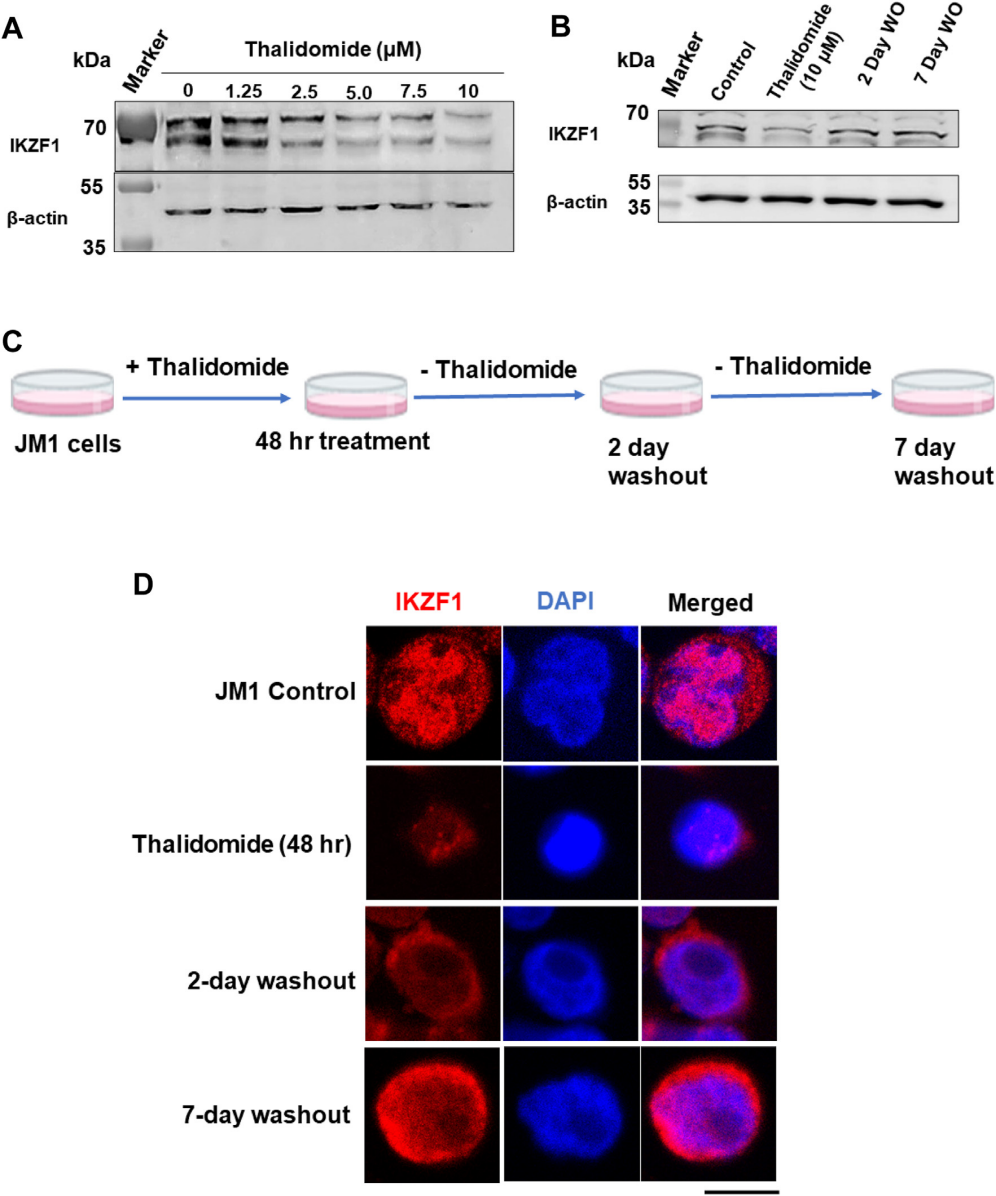
**Thalidomide downregulates the expression of IKZF1.***A*, Western blot analysis of JM1 and JM1 treated with the indicated dose of thalidomide. Beta-actin has been used as a loading control. *B*, Western blot analysis of IKZF1 expression in JM1 cells, JM1 treated with 10 μM thalidomide, thalidomide treated JM1 cells cultured for additional 24 h and 48 h in the absence of thalidomide. Beta-actin has been used as a loading control. *C*, schematic representation of the thalidomide treatment and washout protocol. *D*, representative confocal images of immunofluorescence showing the cytoplasmic mislocalization of IKZF1 in pre-B cell line JM1 upon thalidomide washout. Scale bar represents 4 μm.

Using the JM1 cell line, we next checked the dose response of thalidomide on the expression of IKZF1. We observed a dose-dependent downregulation of IKZF1 in the presence of thalidomide (Fig. 2*A*) without any significant alteration in the viability of JM1 cells after 48 h of treatment (Supplementary Information 1*A*). We then checked if the effects of thalidomide on IKZF1 expression were reversible upon withdrawal of treatment (washout). Our results indicate a progressive recovery of IKZF1 expression 2 days and 7 days after withdrawal of thalidomide (Fig. 2, *B* and *C*). The recovery of IKZF1 expression was about 70.6 percent of the control cells 2 days after washout and almost complete recovery was observed after 7 days post washout (Supplementary Information 1*B*). B-ALL-associated deletion or loss-of-function mutations of *Ikzf1* are known to reduce nuclear IKZF1, thus interfering with its transcriptional regulatory functions (14). Therefore, we tested whether thalidomide could produce such an effect in JM1 cells and if the effect persisted after withdrawal of thalidomide. Comparison of JM1, JM1 treated with thalidomide, and JM1 after thalidomide withdrawal revealed that IKZF1 was majorly retained in the cytoplasm after thalidomide treatment (Fig. 2*D* and Supplementary Information 2*B*). Significantly, IKZF1 was still retained in the cytoplasm after 2 days and 7 days of thalidomide withdrawal (washout) indicating a long-term effect of thalidomide treatment (Fig. 2*D* and Supplementary Information 2, *B* and *C*). Taken together, thalidomide induced a decrease in the expression of IKZF1, and its mistargeting to the cytoplasm could not be completely reversed upon withdrawal of thalidomide.

### Thalidomide treatment and withdrawal mimicked the IK6-mediated increase in stemness and adhesion

**Figure 3:**
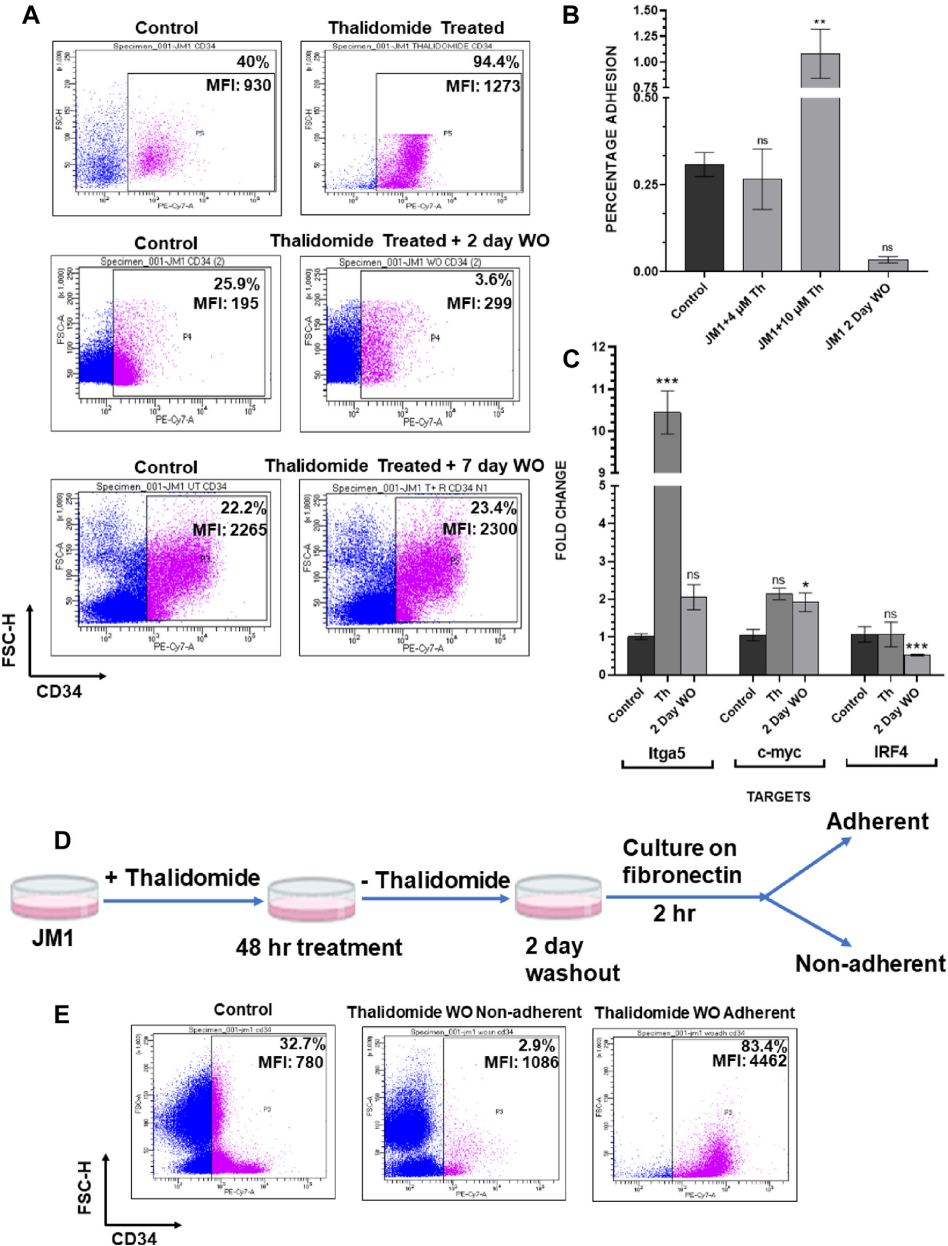
**Thalidomide-dependent increase in stemness and fibronectin adhesion.***A*, expression of CD34 in JM1 control cells and JM1 treated with thalidomide (10 μM), 2 days post thalidomide washout and 7 days post thalidomide washout. The CD34^+^ population is indicated along with the median fluorescence intensity of CD34. The images are representatives of three biological replicates. *B*, JM1 cells treated with 10 μM thalidomide were cultured for an additional 48 h in thalidomide-free media (washout). The cells were cultured in fibronectin-coated plates for 2 h. The JM1 cells in suspension and those adhered to fibronectin were then stained separately for CD34. MFI indicates median fluorescence intensity. *C*, qRT-PCR data showing the fold change in expression of itga5, c-myc, and IRF4 under the indicated conditions. Data has been normalized against the expression of PPIA. Significance measured at ∗*p* < 0.05, ∗∗*p* < 0.01, ∗∗∗*p* < 0.001. *D*, schematic representation of the thalidomide treatment, washout, and further culture on fibronectin. JM1 cells after 2 days of thalidomide washout were plated on a fibronectin-coated plate. After 2 h, cells adhering to fibronectin (adherent) and cells in suspension (non-adherent) were collected. *E*, JM1 cells adhering to fibronectin (thalidomide WO adherent) and cells in suspension (thalidomide WO non-adherent) were collected following the scheme above. Cells were stained for CD34. Flow cytometry analysis was performed and the median fluorescence intensity of CD34 is indicated.

Increased stemness markers are a hallmark of *Ikzf1* mutated B-ALL blasts (10). We checked whether thalidomide treatment could induce similar changes in JM1 cells. Treatment of JM1 with thalidomide caused a significantly high CD34 expression after 48 h of treatment (Fig. 3*A*). Withdrawal of thalidomide decreased the expression of CD34. However, the median fluorescence intensity of CD34 was significantly higher in 2 days post washout cells when compared to control JM1. In contrast, 7 days post washout cells showed comparable CD34 expression as JM1 cells (Fig. 3*A*). This indicated an enhancement of the stemness marker that did not persist after the withdrawal of thalidomide treatment. A similar increase in CD34 expression upon 48 h of thalidomide treatment was observed in three additional B-cell lines – NAMALWA, DAUDI, and NALM6 (Supplementary Information 3). Moreover, persistent upregulation of CD34^+^ population was observed in NAMALWA upon withdrawal of thalidomide treatment for 48 h which was reversed at 7 days of washout (Supplementary Information 3*A*). It is known that deletion of c-myc results in impaired HSC function and progenitor production (15). Strikingly we observed an upregulation of the stemness marker c-myc upon thalidomide treatment in JM1 cells that persisted after withdrawal of treatment (Fig. 3*C*). Additionally, a decrease in the expression of IRF4 which is essential for B-lineage differentiation (Fig. 3*C*) was also observed (16). Along with increased stemness marker, B-ALL blasts carrying *Ikzf1* mutations were reported to show increased adhesion to extracellular matrix proteins such as fibronectin (10). Similar results were observed in JM1 IK6 cells (Fig. 1*B*). Therefore, we analyzed whether thalidomide treatment could induce adhesion to fibronectin. Treatment with thalidomide for 48 h showed a dose-dependent increase in fibronectin adhesion of JM1 cells that was reversible upon withdrawal of thalidomide (Fig. 3*B*). A similar increase in fibronectin adhesion was seen with NAMALWA, DAUDI, and NALM6 after 48 h of thalidomide treatment (Supplementary Information 4). Furthermore, we checked the transcript levels of *Itga5*, a receptor of fibronectin and a known target of IKZF1 repression. Our results showed an increase in the transcript levels of *Itga5* with thalidomide treatment in JM1 cells (Fig. 3*C*) that decreased upon washout. During the development of therapy-induced B-ALL, long-term lenalidomide treatment is expected to alter the differentiation program in a few leukemic stem/progenitor cells. Therefore, we asked whether the cells still adhering to fibronectin after the removal of thalidomide had higher CD34 expression compared to the non-adhered cells (Fig. 3*E*). Our results showed that the adhered cells had a striking increase in CD34 expression (Fig. 3*E*). This indicated that thalidomide treatment caused an irreversible increase in CD34 expression in a sub-population of JM1 cells. Taken together, the observed increase in stemness markers CD34 and c-myc, decrease in B-cell differentiation marker IRF4 and increased fibronectin adhesion indicated a thalidomide-driven reprogramming of the pre-B-cell line JM1.

### Thalidomide-induced alterations in stemness and adhesion are dependent upon IKZF1 expression

**Figure 4:**
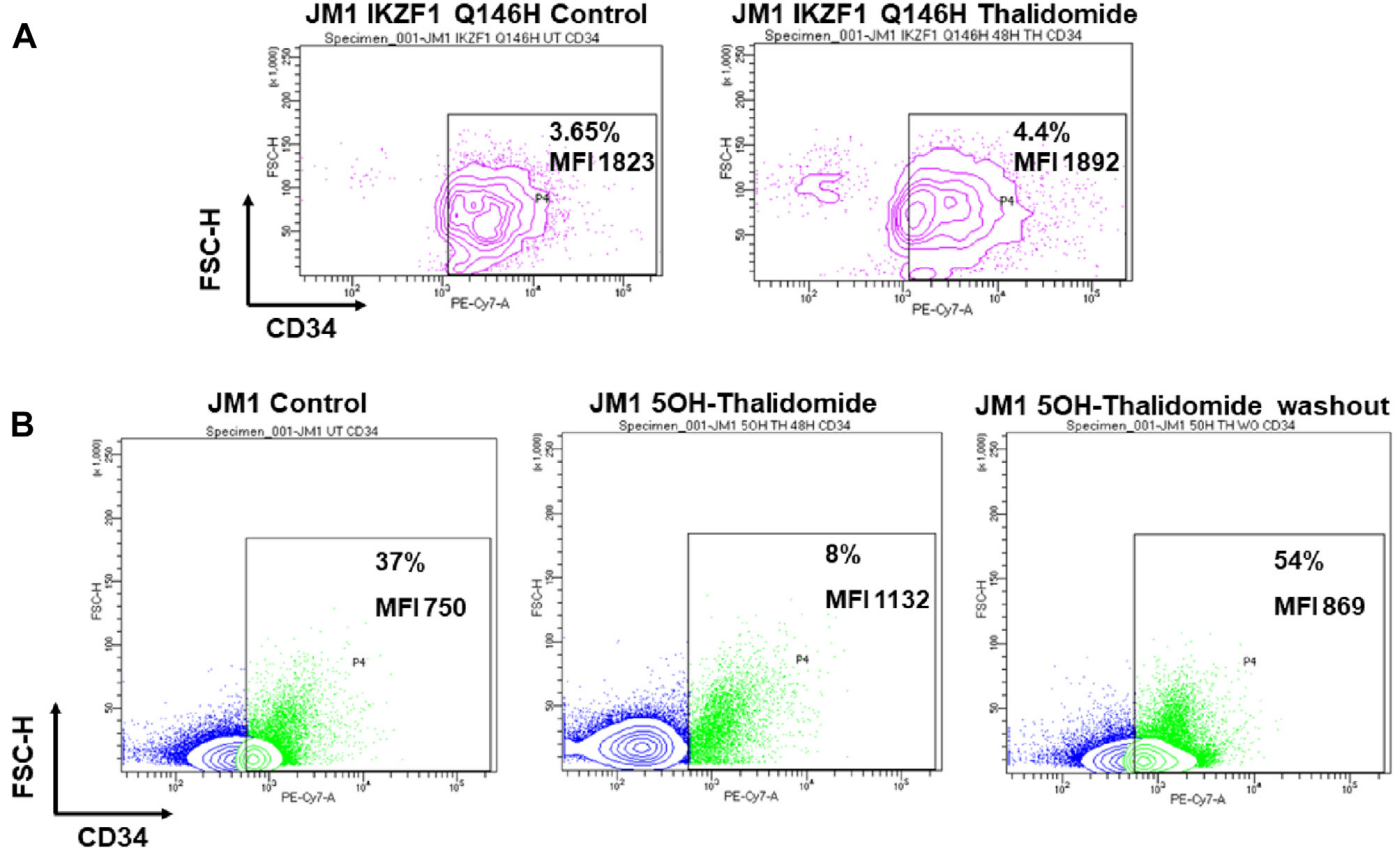
**Thalidomide-dependent alteration in CD34 expression and IKZF1.***A*, flow cytometry data for CD34 levels in JM1 cells electroporated with IKZF1 Q146H and JM1 IKZF1 Q146H cells treated with 10 μM thalidomide. Percentage of parent population and MFI (Median Fluorescence Intensity) is indicated. *B*, flow cytometry data for CD34 levels in control JM1 cells, JM1 treated with the thalidomide analogue 5-OH thalidomide and 2-days washout (WO) of 5-OH treated cells. Percentage of parent population and MFI (Median Fluorescence Intensity) is indicated.

Since thalidomide and its analogs are known to target CRBN E3 ligase to degrade neo-substrates including IKZF1, IKZF3, and SALL4 (6); we checked if the increased stemness upon thalidomide treatment was specific to the downregulation of IKZF1. We electroporated JM1 cells with a thalidomide-resistant IKZF1 Q146H mutant. Treatment of JM1 IKZF1 Q146H with 10 μM thalidomide for 48 h did not produce any change in the percentage of CD34 expressing cells or its median fluorescence intensity (Fig. 4*A*). Finally, we used a derivative of thalidomide (5OH-thalidomide) that does not induce degradation of IKZF1 (17). Western blot confirmed that, unlike lenalidomide, 5OH-thalidomide could not decrease the expression of IKZF1 (Supplementary Information 5A). Treatment with 5OH-thalidomide significantly decreased the percentage of JM1 cells expressing CD34 as compared to control cells which was reversed upon withdrawal of treatment (Fig. 4*B*). It is noteworthy that 5OH-thalidomide treatment withdrawal increased the percentage of CD34^+^ JM1 cells when compared to control, indicating that 5OH-thalidomide may have additional effects on the expression of CD34. Thus, thalidomide-dependent increase in the stemness properties of JM1 cells is dependent upon IKZF1 expression.

### Thalidomide induces a long-term block in B cell differentiation program

**Figure 5:**
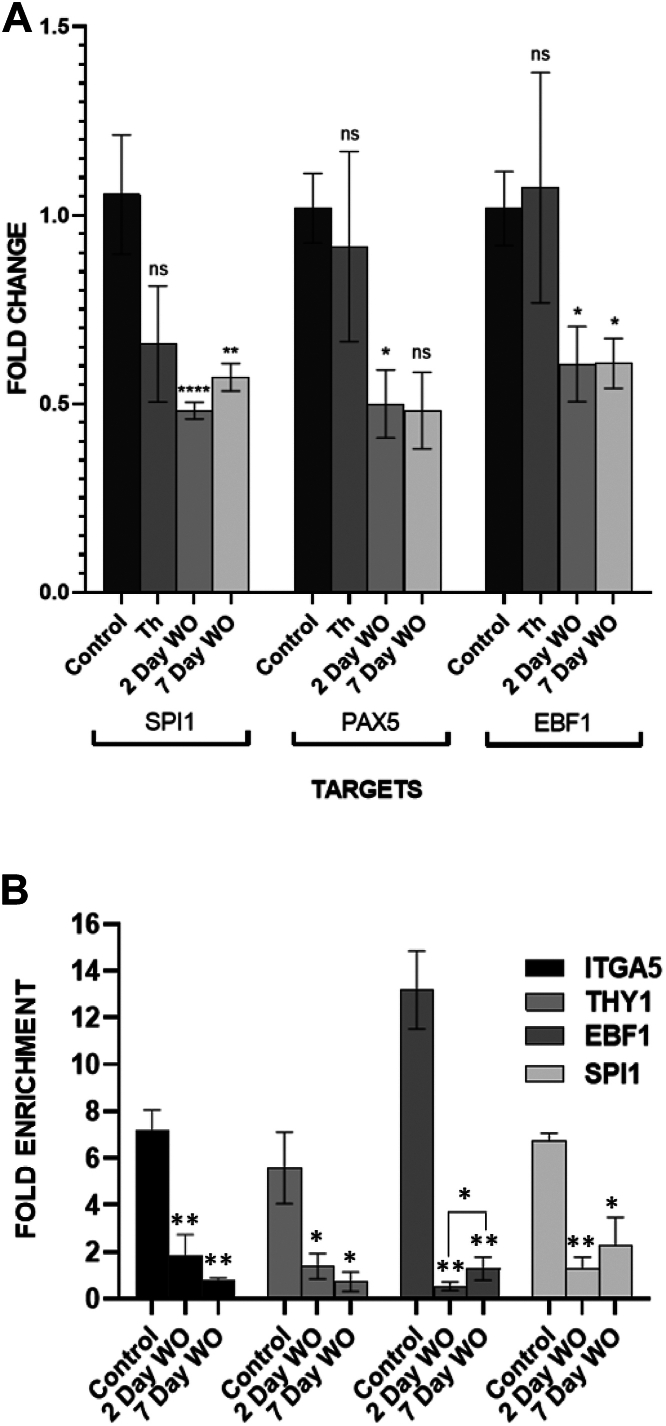
**Thalidomide specifically altered the expression of IKZF1-dependent transcription factors expression.***A*, qPCR analysis of gene expression in JM1 cells treated for 48 h with 10 μM thalidomide, 2 days post washout and 7 days post washout with respect to control JM1 cells. Data has been normalized against the expression of PPIA. Graph indicates mean ± SEM of three biological replicates. *B*, chromatin immunoprecipitation of IKZF1 bound promoter regions corresponding to the genes *itga5, thy1, ebf1, and spi1*. qPCR data shows fold enrichment of the promoter regions in 2 days and 7 days post thalidomide washout conditions when compared to JM1 control (untreated) cells. Significance measured using student’s *t* test ∗*p* < 0.05, ∗∗*p* < 0.01, ∗∗∗*p* < 0.001, ∗∗∗∗*p* < 0.0001.

Deletion or loss of function mutations of *Ikzf1* are known to alter the profile of B-cell-specific transcription factors such as Pax5 and EBF1 (18). Therefore, we used JM1 cells transduced with the dominant negative IKZF1 construct (IK6) that is a product of the exon 4 to 7 deletion mutation of *Ikzf1* commonly detected in B-ALL patients. We observed downregulation of Pax5, EBF1, and Spi1 in the presence of IK6 (Fig. 1*C*). Treatment of JM1 cells with thalidomide for 48 h did not produce any significant alterations in the expression of these transcription factors (Fig. 5*A*). Strikingly, the expression of Pax5, Spi1, and EBF1 was significantly downregulated 2 days post withdrawal (washout) of thalidomide treatment (Fig. 5*A*). This downregulation of B-cell specification factors persisted 7 days post thalidomide washout (Fig. 5*A*) indicated a long-term effect of thalidomide on the expression of B-cell lineage transcription factors. To evaluate the effect of thalidomide on the chromatin occupancy of IKZF1, we performed chromatin immunoprecipitation of IKZF1 bound nuclear DNA in JM1 control cells and JM1 cells 2 days and 7 days post thalidomide washout. Unlike JM1 cells, we failed to observe enrichment of the promoter regions of IKZF1 targets *Itga5* and *Thy1* in 2 days and 7 days post thalidomide recovery samples (Fig. 5*B*). Our results were in accordance with the IKZF1 cytoplasmic localization observed in the thalidomide recovery samples (Fig. 2*D*). Strikingly, the chromatin occupancy of IKZF1 at the promoter regions of EBF1 and Spi1 (PU.1) significantly decreased in the washout samples when compared to JM1 control (Fig. 5*B*). This indicated a long-term effect of thalidomide on the B-cell specifying transcription factors, explaining the downregulation in their expression specifically after thalidomide withdrawal. Pax5 promoter region was not found to be bound by IKZF1 in JM1 cells or in any of the thalidomide conditions indicating that Pax5 was not a direct transcriptional target of IKZF1. Thus, thalidomide irreversibly altered the transcription factor network which is crucial to the developing B-cell lineage resulting in a prolonged reduction in the expression of B cell lineage transcription factors.

## Discussion

Patients with MM often receive high-dose chemotherapy with autologous hematopoietic cell transplant (HCT) followed by long-term maintenance therapy with lenalidomide. Treatment with lenalidomide improves the survival of MM patients but is accompanied by an increased risk of secondary malignancies. These secondary malignancies are clonally distinct from the primary tumor and manifest as therapy-related B-ALL (3, 4, 19). However, the molecular mechanism by which the therapy-related B-ALL arises remains unexplored. Lenalidomide itself is an analogue of thalidomide, and like thalidomide is known to degrade IKZF1 and IKZF3 in multiple myeloma cells. This was shown to be necessary for the drug’s antileukemic activity (20). Interestingly, IKZF1 is a crucial transcription factor whose absence blocks B cell differentiation in the early stages of the lineage whereby the pro and the pre-B cells adhere strongly to the bone marrow matrix (8, 9). B-ALL blasts carrying *Ikzf1* deletion or loss of function mutations were observed to have an enhanced expression of stemness factors like CD34 and cell adhesion (9, 10). Finally, lenalidomide was found to degrade both IKZF1 and IKZF3 in the context of multiple myeloma (20). Therefore, we asked if thalidomide-induced IKZF1 degradation could lead to alterations in B cell differentiation program.

We used a pre-B ALL cell line JM1 transduced with the dominant negative IKZF1 mutant IK6 (21). The resulting JM1 IK6 recapitulated the B-ALL blast phenotype of increased stemness and cell adhesion. Next, we used the JM1 cells to check the effects of thalidomide treatment on the B cell differentiation program. We observed that thalidomide induced an increase in CD34 expression concomitant to the reduction of IKZF1. Increased CD34 expression persisted in a sub-population of fibronectin adherent JM1 cells 2 days post-withdrawal of thalidomide. Notably, the half-life of recovery of proteins engineered with IKZF1-like degron following thalidomide withdrawal was reported to be ∼4 h (22). Therefore, the reduced expression of IKZF1 2 days post thalidomide washout was unlikely to be a consequence of residual thalidomide remaining inside the cells. Importantly, thalidomide-dependent increase in CD34 expression was seen in three other B cell lines indicating this to be a general mechanism in B cells. To further understand if the effects of thalidomide treatment were persistent, we checked the localization of IKZF1. Surprisingly, IKZF1 was found to persist in the cytoplasm 7 days post washout indicating that thalidomide treatment might interfere with IKZF1-dependent transcriptional regulation. Indeed, thalidomide treatment increased the expression of *Itga5* and thus increased fibronectin adhesion of JM1 cells. However, *Itga5* expression quickly reduced to basal levels post thalidomide washout.

B-cell differentiation is guided by a network of lymphoid-specific transcription factors (18). We observed that thalidomide treatment for 48 h did not cause any significant change in the expression of key B-cell lineage transcription factors Pax5, EBF1, and Spi1 (PU.1). Surprisingly, 2 days and 7 days post thalidomide washout samples showed a significant reduction in the expression of Pax5, EBF1, and Spi1. Since EBF1 and Spi1 were known to be transcriptionally regulated by IKZF1 (23, 24, 25), we checked if thalidomide could cause a long-term alteration in the chromatin occupancy of IKZF1 at the promoter loci of Pax5, Spi1, and EBF1. Indeed, IKZF1 enrichment was reduced at the promoters of Spi1 and EBF1 at day 2 and 7 post-thalidomide washout. This indicated a long-term downregulation of IKZF1-dependent transcription in cells pre-treated with thalidomide. Finally, using thalidomide-resistant IKZF1 Q146H and IKZF1 non-targeting analog 5OH-thalidomide, we showed that IKZF1 was necessary and sufficient to cause thalidomide-induced increase in CD34 expression.

In conclusion, treatment with thalidomide irreversibly altered the B-cell transcription factor network due to the cytoplasmic mislocalization of IKZF1. Such downregulation in the B cell transcription factors created a persistent block in the B-cell differentiation program. It is possible that the presence of p53 mutations as seen in multiple myeloma patients developing therapy-related B-ALL (3, 4), enhanced the proliferative capacity of these cells to develop into B-ALL blasts. Our results also explain why early detection of B-ALL-like blasts followed by cessation of lenalidomide therapy was sufficient for at least one MM patient to achieve spontaneous remission from therapy-related B-ALL (4). Thus, our data provides a molecular mechanism for the development of therapy-related B-ALL blasts in patients with MM receiving thalidomide analog.

## Experimental procedures

### Cell culture

JM1, NALM6, DAUDI, and NAMALWA cells were maintained in RPMI 1640 (HIMEDIA, Cat. Number AL199A) supplemented with 10% FBS (GIBCO, Cat. Number A5256801) and 5% penicillin/streptomycin (HIMEDIA, Cat. Number A018). The cell lines were obtained from NCCS, Pune India, and validated at the source. Similarly, HEK 293T cells were maintained in complete DMEM (HIMEDIA, AL007A). All cell lines were maintained at 37 °C and 5% CO_2_.

### Transduction

In a lipofectamine (Invitrogen, Cat. Number 11668030) mediated transfection, HEK 293T cells were co-transfected with pCMV and pMD2G constructs along with either MSCV-IKZF1-IRES-RFP or MSCV-IK6-IRES-RFP. These plasmids were kindly gifted to us by Dr Charles G. Mullighan. Virus supernatants were collected and JM1 cells were infected with the retrovirus according to the protocol detailed in Supplementary Methods. RFP^+^ cells were sorted on BD FACS ARIA III.

### Thalidomide treatment

Cells were treated with 4 μM and 10 μM Thalidomide (Cayman Chemical, CAS Number 50-35-1) dissolved in DMSO (Sigma-Aldrich Cat. Number D1418), for a period of 48 h. For experiments involving washout of thalidomide, 48-h thalidomide-treated cells were washed in PBS and subsequently grown in fresh media for another 2 days for 2-day washout (WO) and 7 days for 7-days washout (WO) studies.

### RNA Isolation, cDNA synthesis, and qPCR

Cells were pelleted down and washed with PBS (MP Biomedicals, Cat. Number 2810305). RNA was isolated using TRIzol (Invitrogen, Cat. Number 15596026) following the manufacturer’s protocol. Complementary DNA (cDNA) was prepared from the isolated RNA according to kit specifications (Super Rev. Transcriptase MuLV Kit – BioBharati Lifesciences, Cat. Number BB-E0042). qPCR reactions were performed as per kit specifications (iTaq Universal SYBR Green Supermix – Bio-Rad, Cat. Number 1725124) in the BIORAD CFX96 Real Time detection system. Fold change was calculated using the delta-delta Ct method against the housekeeping gene PPIA and HPRT. Primers used in the reaction may be found in Supplementary Methods.

### Flow cytometry

Cells were pelleted down at 1000 RCF for 5 min at 4 °C. This was followed by a PBS wash, where the cells were resuspended in 1X PBS and spun down at 1000 RCF for 5 min at 4 °C. The cells were then incubated with antibodies against CD19 APC (BD Biosciences, Cat. Number 340437), CD10 APC (Beckman Coulter Life Sciences, Cat. Number IM3633), and CD34 PE-Cy5.5 (Invitrogen, Cat. Number CD34-581-18) for 30 min in the dark. The cells were washed and finally resuspended in PBS and transferred to FACS tubes for flow cytometry (BD Fortessa X20). Additionally, CD34 PE-Cy7A (BD Biosciences, Cat. Number 560710) was used for thalidomide experiments.

### Fibronectin adhesion assay

Wells of 96 well plates were coated with fibronectin solution (1 μg/ml) (Sigma-Aldrich, Cat. Number F4759) and incubated at 37 °C for 30 min. The excess solution was removed, and the plates were stored at 4 °C for subsequent usage. Cells were seeded onto the fibronectin-coated plates in triplicates and incubated at 37 °C for 2 h. The wells were then imaged at 4x magnification and washed with 1X PBS three times. Post-wash images were then taken at 4x magnification. Cells in pre-wash and post-wash images were counted using the ImageJ processing program. The percentage of adhesion was calculated by the number of cells adhered post wash by the total number of cells in the wells prewash. For adhesion assays with thalidomide treatment, cells were treated with 4 μM and 10 μM thalidomide and incubated for 48 h. Treated cells were then seeded onto fibronectin coated 96 well plates and the protocol for adhesion assay was followed from thereon.

### Immunofluorescence and microscopy

JM1 control or JM1 cells under different thalidomide conditions were fixed with 4% formaldehyde. Cells were permeabilized and stained with anti-IKZF1 antibody (Abclonal Catalog Number A1850). This was followed by incubation with a secondary anti-rabbit tagged with Alexa 568. Details of the protocol may be found in (26). Cells were washed and mounted with mounting media containing DAPI. Images were acquired in widefield mode under a fluorescence microscope using a 20X objective lens. Slides were also imaged under a Leica SP8 confocal microscope under a 63X oil objective.

### Chromatin immunoprecipitation

JM1 cells were harvested and cross-linked with 1% formaldehyde followed by the shearing of chromatin into fragments (200–800 bp). 25 μg of chromatin was incubated with anti-IKZF1 antibody (3 μg) along with Peirce A/G beads (ThermoFisher) overnight with gentle rocking at 4 °C. Immunoprecipitated components were collected, quantified, and processed by qRT-PCR using specific primers (Supplementary Methods) to check the promoter occupancy of IKZF1 on different genes.

### Western blot analysis

Briefly, 5 × 10^5^ JM1 cells were treated with thalidomide (10 μM) for 48 h. In drug recovery conditions, cells were washed and replaced with fresh media without thalidomide (Th-washout). Cells were cultured for an additional 2 days and 7 days in a humidified 5% CO_2_ incubator. Total protein was extracted using RIPA buffer and quantified by BCA Kit (Thermofisher). An equal amount of protein (30 μg) was separated on 12% SDS-PAGE and transferred onto a nitrocellulose membrane. Further, membranes were incubated with an anti-IKZF1 antibody (Abclonal) and probed with an HRP-conjugated anti-rabbit secondary antibody. The blots were developed using an enhanced chemiluminescent (ECL) detection kit (Thermo Scientific). β-actin (Cell Signaling Technology Inc) was used as a loading control.

### Statistical analysis

All data are presented as mean value ± standard error of mean. Student’s *t* test was performed and *p* values less than or equal to 0.05 were considered significant (∗*p* ≤ 0.05, ∗∗*p* ≤ 0.01, ∗∗∗*p* ≤ 0.001, ∗∗∗∗*p* ≤ 0.0001).

## Data availability

The data generated in this study are available upon reasonable request from the corresponding authors.

## Supporting information

This article contains supporting information.

## Conflict of interest

The authors declare that they have no conflicts of interest with the contents of this article.
