# Supplementary material for: A pre-B acute lymphoblastic leukemia cell line model reveals the mechanism of thalidomide therapy-related B-cell leukemogenesis
Source: J Biol Chem. 2024 Jul 17;300(8):107578. doi: 10.1016/j.jbc.2024.107578 (PMC11367411; doi:10.1016/j.jbc.2024.107578)
Supplement: Supplementary Methods [file mmc1.docx]

**SUPPLEMENTARY METHODS**

**PRIMERS**

Primers used in this study for qPCR:

| Gene Name | Forward(FP), Reverse (RP) | Primer Sequence (5’ to 3’) | Product Length |
| --- | --- | --- | --- |
| AJUBA | FP | AGGCCAGGGAGGACTACTTC | 135 |
|  | RP | GCAAAGTTCGCCCACAAGAG |  |
| EBF1 | FP | CCTCTTATCTGGAACATGCAGC | 179 |
|  | RP | CACACGGATGGCATGAGGAG |  |
| CD52 | FP | GCCTCCTGGTTATGGTACAGATAC | 94 |
|  | RP | CCGCTTATGTTGCTGGATGC |  |
| TIAM1 | FP | CCCATCTCCTCAGGACTCCA | 199 |
|  | RP | AAGCACGTCAAGCTCATCCT1 |  |
| ITGA5 | FP | TGCCTCCCTCACCATCTTC | 171 |
|  | RP | TGCTTCTGCCAGTCCAGC |  |
| PAX5 | FP | CCAGCAGGACAGGACATGG | 121 |
|  | RP | CAGGGCCTGACACCTTGATG |  |
| SPI1 | FP | ATCCTGAGGGGCTCTGCATT | 125 |
|  | RP | AGGTCTTCTGATGGAGGGGG |  |
| PPIA | FP | GCCGAGGAAAACCGTGTACT | 109 |
|  | RP | TGTCTGCAAACAGCTCAAAGG |  |
| HPRT | FP | CCTGGCGTCGTGATTAGTGA | 137 |
|  | RP | CGAGCAAGACGTTCAGTCCT |  |

Primers used in this study for ChIP-qPCR:

| Gene Name | Forward(FP), Reverse (RP) | Primer Sequence (5’ to 3’) | Product Length |
| --- | --- | --- | --- |
| ITGA5 | FP | GACCCCAAAGTCTCCTCCCT | 140 |
|  | RP | ACGTAAACGCTCGGAAAACG |  |
| EBF1 | FP | CGTCCTCCTCATCTACACGC | 129 |
|  | RP | TGCCGGCTTTGCTACTTCAA |  |
| THY1 | FP | ACAGCTGGAAGGTTCTTCTGG | 87 |
|  | RP | CCGCTTATGTTGCTGGATGC |  |
| SPI1 | FP | CCCAGGCAAGGGAAGTTTGT | 160 |
|  | RP | ACAGGAGTCTAGGGAGGCTG |  |

**Transduction**

In a lipofectamine (Invitrogen, Cat. Number 11668030) mediated transfection, HEK 293T cells were co-transfected with pCMV and pMD2G constructs along with either MSCV-IKZF1-IRES-RFP or MSCV-IK6-IRES-RFP. These plasmids were kindly grifted to us by Dr. Charles G. Mullighan. Six hours post transfection, incomplete DMEM was replaced with complete DMEM (10% FBS, 5% pen/strep). 12 hours after the media change, cells were cultured in low serum media (DMEM supplemented with 2% FBS) for 24 hours. Following this, viruses were concentrated using centrifugal filter units (Merck, Amicon® Ultra-4 Centrifugal Filter Unit, Cat. Number UFC801096) at 3,000 RCF for 30 minutes at 4⁰C. For the purpose of transduction, JM1 cells were cultured overnight in starvation media (RPMI 1640 supplemented with 1% FBS). Cells were then incubated in complete RPMI (RPMI 1640 supplemented with 10% FBS) for an hour. Cells were pelleted down and viruses were added to the pellet and spun at 800 RCF for 30 minutes. The viruses and cells were transferred to T25 flasks where virus infection was allowed for 6 hours, followed by a media change (RPMI 1640 supplemented with 10% FBS). Successfully transduced cells were sorted (BD FACSAria II) based on RFP expression and subsequently cultured and maintained for further experimentation.

**Cell viability assay**

MTT assay was carried out to check the effect of thalidomide on JM1 cell viability. 1 ×10^4^ JM1 cells were seeded in a 96 well culture plate in complete RPMI media and kept in a 5 % CO_2_ incubator at 37⁰C. Different concentrations of thalidomide (ranging from 0-10 µM) was added to each well and incubated for 48 hours. Following treatment, the culture media was removed and cells were again incubated with MTT (0.5 mg/mL) at 37⁰C for 4 hours. The formed blue formazan crystals were dissolved by adding DMSO (100µl) and absorbance was taken at 595 nm.

The % of viable cells was calculated using the following equation:

% Cell Viability = [Absorbance in the treated group]/ [Absorbance in the untreated group] × 100

**Nuclear cytoplasmic fractionation**

Briefly, cells were washed with ice-cold PBS and harvested at 300 × g for 5 min at 4 °C. Cytosol was extracted using ice-cold cytosol extraction buffer (50 mM Tris-HCl, pH 7.5, 10 mM NaCl, 2 mM EDTA, 1 mM PMSF, 1× protease inhibitor cocktail). The cells were incubated in the cytosolic extraction buffer for 10 min on ice. 0.25% NP-40 was then added, and the cells were vortexed for 5 seconds before being pelleted at 800 × g for 5 min. The supernatant was further centrifuged to remove contaminating cell remnants and the resulting supernatant was collected as the cytosolic fraction. The nuclear pellet was washed thrice with cytosolic extraction buffer to remove any residual cytosolic fraction and then suspended in nuclear extraction buffer (50 mM Tris-HCl, pH 7.5, 400 mM NaCl, 2 mM EDTA, 1 mM PMSF, 1× protease inhibitor cocktail), incubated on ice for 10 min with intermittent vortexing followed by centrifugation at 10,000 × g for 15 min at 4 °C. The resulting supernatant was collected as the nuclear fraction. Western blot analysis was done to detect IKZF1 in the cytosolic and nuclear fractions. GAPDH was used as the cytosolic control and Histone H3 as nuclear control.
