# Supplementary material for: A pre-B acute lymphoblastic leukemia cell line model reveals the mechanism of thalidomide therapy-related B-cell leukemogenesis
Source: J Biol Chem. 2024 Jul 17;300(8):107578. doi: 10.1016/j.jbc.2024.107578 (PMC11367411; doi:10.1016/j.jbc.2024.107578)
Supplement: Supplementary Information [file mmc2.pdf]

SUPPLEMENTARY INFORMATION 1

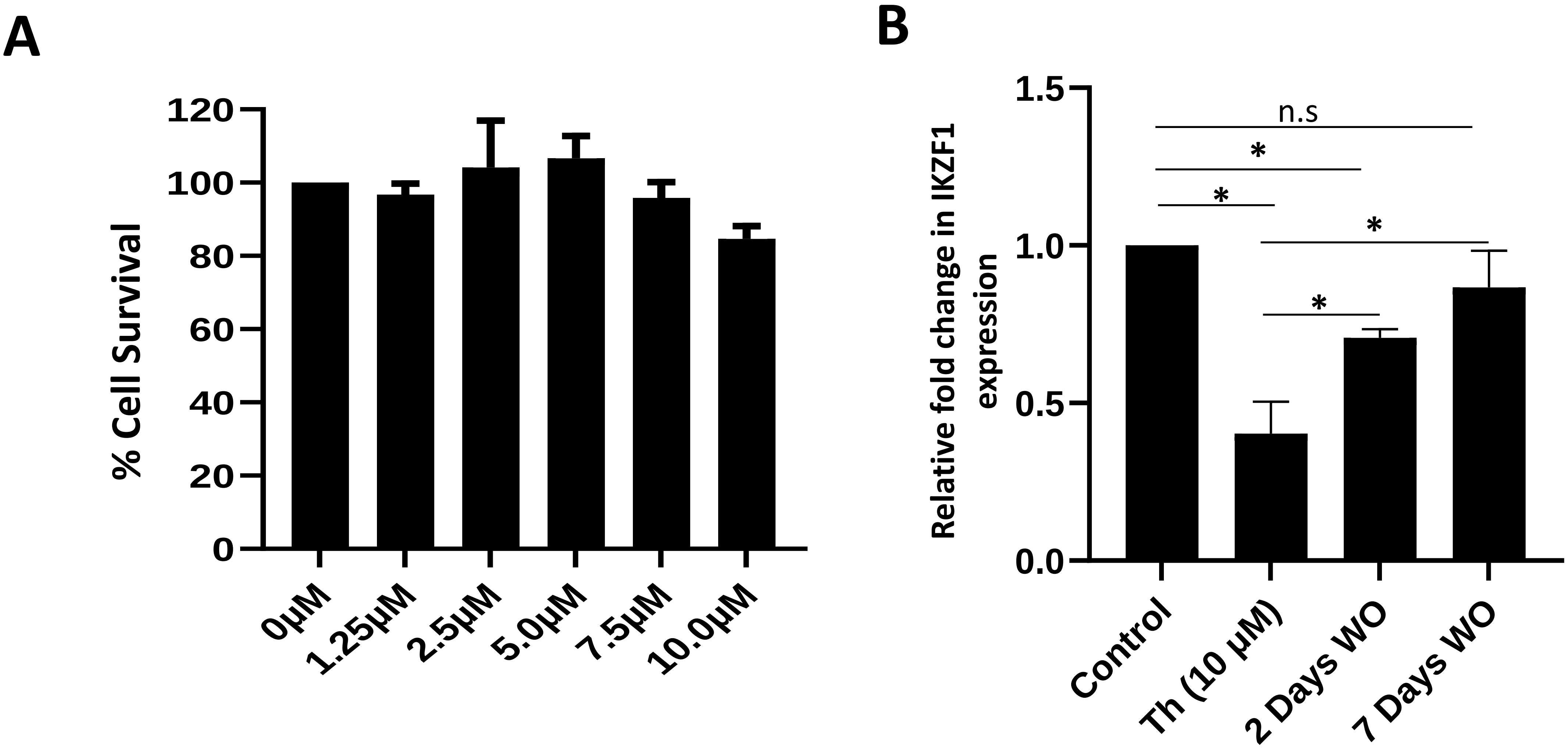

SUPPLEMENTARY INFORMATION 2

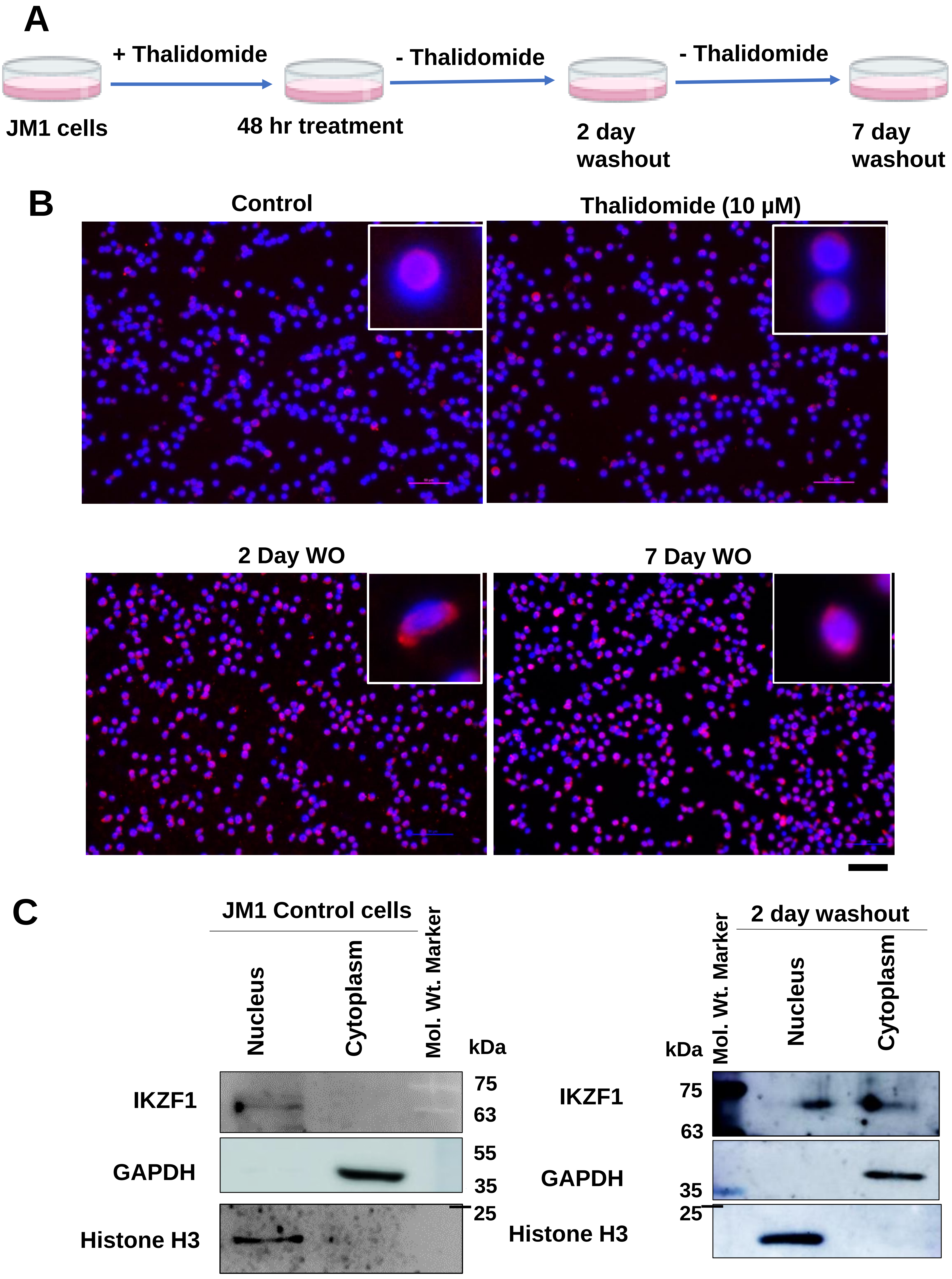

SUPPLEMENTARY INFORMATION 3

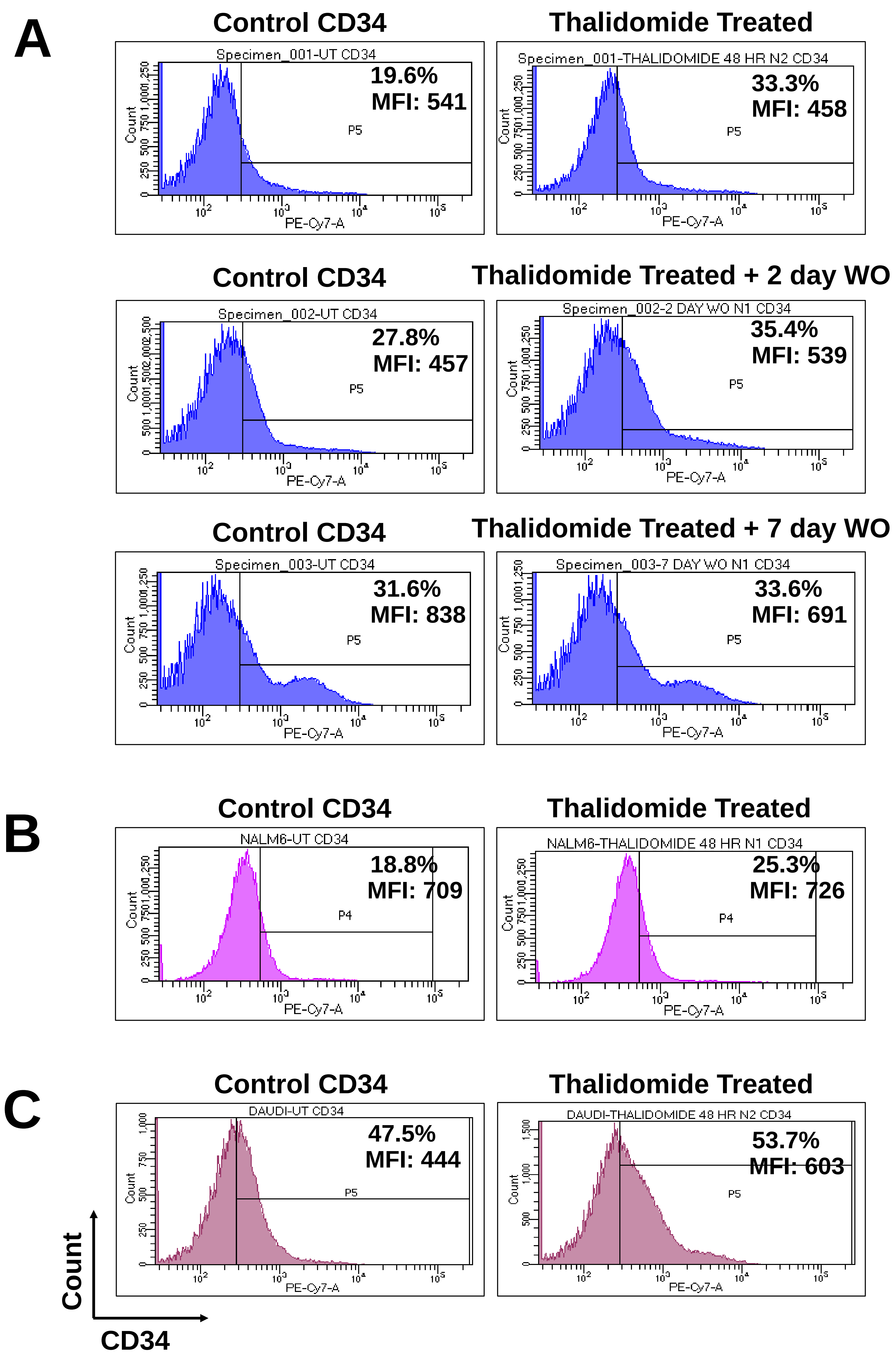

SUPPLEMENTARY INFORMATION 4

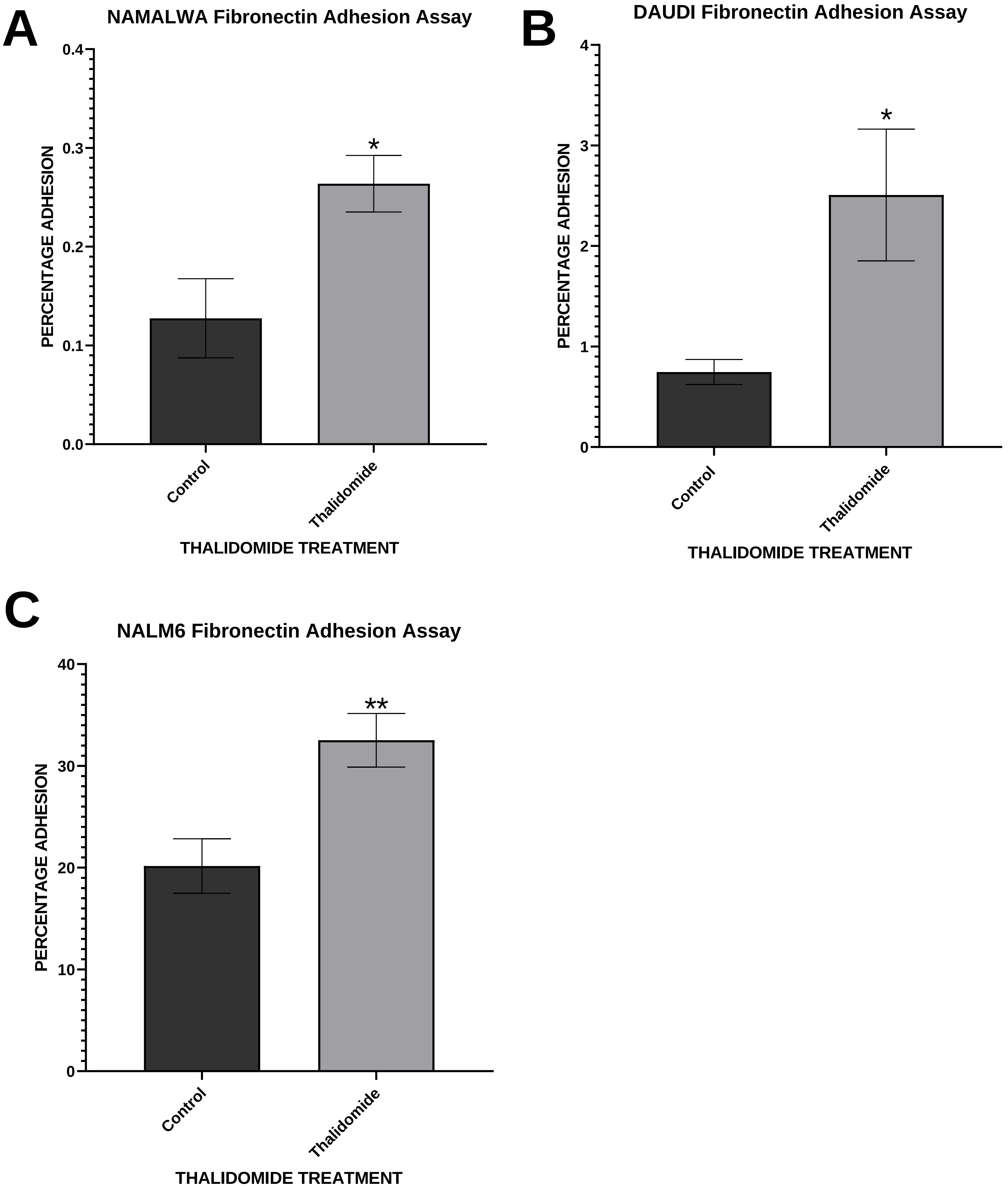

SUPPLEMENTARY INFORMATION 5

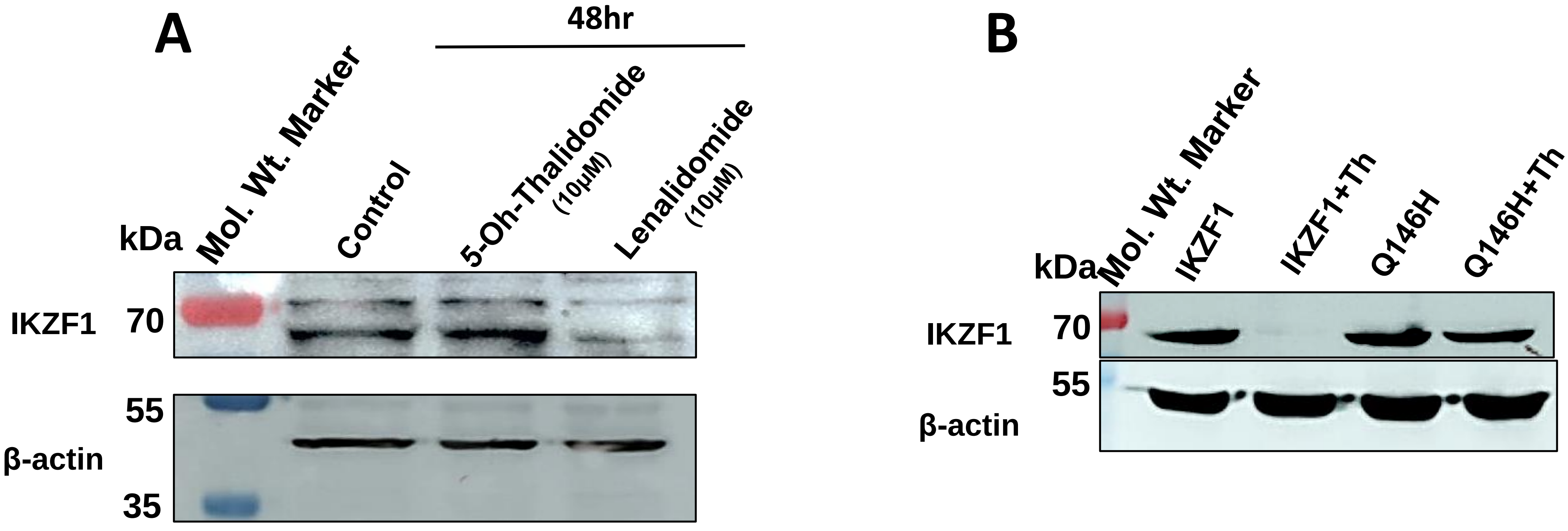

SUPPLEMENTARY LEGENGS:

Supplementary Information 1. (A) MTT assay was carried out to check the effect of thalidomide on the cell viability of JM1 cells. (B) JM1 cells treated with thalidomide and IKZF1 expression were checked by western blotting at the indicated conditions. The densitometric analysis of the expression of IKZF1 normalized to beta actin from three biological replicates has been plotted (Mean ± sem). Significance measured using student's t-test *$p < 0.05$.

Supplementary Information 2. (A) Schematic representation of the thalidomide treatment and washout protocol. (B) Representative images of immunofluorescence showing the cytoplasmic mislocalization of IKZF1 in pre-B cell line JM1 upon thalidomide washout. Scale bar represents 50 µm. (C) JM1 control cells and JM1 after 2 days of thalidomide washout were subjected to nuclear and cytoplasmic fractionation. The fractions were probed for IKZF1, GAPDH (cytoplasmic control) and Histone H3 (nuclear control).

Supplementary Information 3. (A) NAMALWA cells were treated with 10 µM thalidomide for 48 hr followed by culture in thalidomide free media for an additional 7 days. The expression of CD34 was assessed by flow cytometer in control cells, cells after 48 hr thalidomide treatment, cells after 2 day thalidomide withdrawal and cells after 7 day thalidomide withdrawal. (B) CD34 expression in NALM6 control cells and cells after 48 hr of thalidomide treatment. (C) CD34 expression in DAUDI control cells and cells after 48 hr of thalidomide treatment.

Supplementary Information 4. Fibronectin adhesion assay was performed using control cells and cells after 48 hr of thalidomide treatment in (A) NAMALWA (B) DAUDI and (C) NALM6. The percentage of adherent cells is indicated. Significance measured using student's t-test for n=3, *$p < 0.05$, **$p < 0.01$

Supplementary Information 5. (A) IKZF1 expression was checked in JM1 cells treated with 5-OH thalidomide and lenalidomide. (B) Cells expressing IKZF1 and IKZF1-Q146H constructs were treated with/without thalidomide for 48 hr. Western blotting was performed to check the expression of the IKZF1 and IKZF1 Q146H.
